# Differential regulation of CCL-11/eotaxin-1 and CXCL-8/IL-8 by Gram-positive and Gram-negative bacteria in human airway smooth muscle cells

**DOI:** 10.1186/1465-9921-9-30

**Published:** 2008-04-01

**Authors:** Razao Issa, Rosalinda Sorrentino, Maria B Sukkar, Shiranee Sriskandan, Kian Fan Chung, Jane A Mitchell

**Affiliations:** 1 Experimental Studies, Airway Disease Section, National Heart & Lung Institute, Imperial College London, London, SW3 6LY, UK; 2 Cardiothoracic Pharmacology, Unit of Critical Care Medicine, National Heart and Lung Institute, Imperial College London, London, SW3 6LY, UK; 3 Department of Infectious Diseases & Immunity, Division of Investigative Science, Hammersmith Campus, Imperial College London, London, W12 ONN, UK; 4 Novartis, Horsham, West Sussex, RH12 5AB, UK

## Abstract

**Background:**

Bacterial infections are a cause of exacerbation of airway disease. Airway smooth muscle cells (ASMC) are a source of inflammatory cytokines/chemokines that may propagate local airway inflammatory responses. We hypothesize that bacteria and bacterial products could induce cytokine/chemokine release from ASMC.

**Methods:**

Human ASMC were grown in culture and treated with whole bacteria or pathogen associated molecular patterns (PAMPs) for 24 or 48 h. The release of eotaxin-1, CXCL-8 or GMCSF was measured by ELISA.

**Results:**

Gram-negative *E. coli *or Gram-positive *S. aureus *increased the release of CXCL-8, as did IL-1β, LPS, FSL-1 and Pam_3_CSK4, whereas FK565, MODLys18 or Poly I:C did not. *E. coli *inhibited eotaxin-1 release under control conditions and after stimulation with IL-1β. *S. aureus *tended to inhibit eotaxin-1 release stimulated with IL-1β. *E. coli *or LPS, but not *S. aureus*, induced the release of GMCSF.

**Conclusion:**

Gram-positive or Gram-negative bacteria activate human ASMC to release CXCL-8. By contrast Gram-negative bacteria inhibited the release of eotaxin-1 from human ASMCs. *E. coli*, but not *S. aureus *induced GMCSF release from cells.

Our findings that ASMC can respond directly to Gram-negative and Gram-positive bacteria by releasing the neutrophil selective chemokine, CXCL-8, is consistent with what we know about the role of neutrophil recruitment in bacterial infections in the lung. Our findings that bacteria inhibit the release of the eosinophil selective chemokine, eotaxin-1 may help to explain the mechanisms by which bacterial immunotherapy reduces allergic inflammation in the lung.

## Background

In diseases such as asthma, the airway smooth muscle fulfills a contractile role. However, airway smooth muscle cells (ASMC) can also acquire a secretory phenotype with the ability to release inflammatory mediators including cytokines, chemokines [1-4], and lipid hormones [5] when stimulated with cytokines or oxidants [6,7].

The role of bacteria in airway inflammation is currently the subject of investigation. For example, bacterial infections may exacerbate asthmatic symptoms and eradication therapy appears to provide some therapeutic benefits [8,9]. On the other hand, low level inoculation immunotherapy with bacterial components may provide therapeutic benefit in the treatment of allergic asthma, presumably by redressing the balance between type1 (Th1; T helper 1) and type 2 inflammatory pathways [10]. In addition to asthma, it has been recognized for some time that bacterial infections are the underlying cause of exacerbations in some patients with COPD [11]. It is therefore important that we understand how bacteria and other pathogens are sensed by airway cells. Human ASMCs express Toll like receptors (TLRs), including TLR2 and TLR4 [12,13]. Stimulation of ASMCs with pathogen associated molecular patterns (PAMPs) including LPS induce either low or undetectable levels of cytokines [12-14], although the sensing of LPS, for example, by ASMCs can be enhanced dramatically by co-culture with monocytes [12]. However the effects of whole bacteria on release of cytokines from human ASMCs has not previously been studied. Moreover, the effect of bacteria or associated PAMPs on the eosinophil- selective chemokines released by airway smooth muscle is not completely understood.

In the current study we have used whole Gram-negative *Escherichia coli *and Gram-positive *Staphylococcus aureus *as model organisms, as well as a range of well characterized PAMPs, to compare directly their effects on the release of CXCL-8, which recruits neutrophils, and eotaxin-1, which recruits eosinophils. The level of GMCSF, which is active on both eosinophils or neutrophils was also measured.

## Methods

### Materials

Eotaxin-1, CXCL-8 and GMCSF enzyme-linked immunosorbent assays (ELISA) DuoSet kits were purchased from R&D Systems (Abingdon, UK). The cell culture plastic ware was purchased from Falcon Labware (Becton Dickinson, Oxford, UK). The Pam_3_CSK4, LPS and FSL-1 were purchased from Axxora (UK) Ltd (Nottingham, U.K). All other tissue culture reagents and chemicals were obtained from Sigma (Poole, UK) unless otherwise stated.

### Human airway smooth muscle cell isolation and culture

Main lobar bronchi obtained from patients undergoing lung resection for carcinoma of the bronchus were used for dissecting human ASMC, as previously described [4]. Cells were maintained in Dulbecco's modified Eagle's medium (DMEM) containing 10% foetal calf serum FCS supplemented with sodium pyruvate, L-glutamine (2 mM), non-essential amino acids (1:100), penicillin (100 U ml^-1^)/streptomycin (100 μg ml^-1^) and amphotericin B (1.5 μg ml^-1^) in a humidified atmosphere at 37°C in air/CO_2 _(95:5 % vol/vol). Confluent cells were passaged with 0.25% trypsin and 1 mM EDTA. Cells at passages 3–7 from 6 different donors were used in the studies described below. In pilot studies ASMC were initially characterized by positive immunostaining for calponin, smooth muscle α-actin and myosin heavy chain. There after they were characterized by their typical morphology and phenotype.

### Preparation of *E. coli *and *S. aureus*

*E. coli*, reference strain 0111.B4, and *S. aureus *H380 were used. The *S. aureus *H380 was isolated from clinical blood culture and stored frozen in 15% glycerol. This was streaked onto agar plates prior to inoculation of single colonies into RPMI-1640 medium with 10% FCS and glutamine at 37°C overnight. The bacteria were pelleted by centrifugation at 800 × *g *then washed in sterile saline twice. The pellets were re-suspended in sterile saline. In order to quantify the cell density, aliquots of the bacterial suspension were serially diluted and plated onto agar. The bacteria in the bacterial suspensions were killed by heat treatment for 45 min at 80°C. The resultant suspensions were plated to confirm the sterility. Suspensions were adjusted to 10^10^-10^12 ^colony-forming units (CFUml^-1^) and then frozen with 20% glycerol in aliquots before use in cell culture experiments.

### ELISAs

Measurement of GMCSF, CXCL-8 and eotaxin-1 in culture supernatants was performed using ELISA DuoSets according to manufactures' instructions (R&D Systems, Abingdon, UK). Supernatants were stored at -80°C prior to assay.

### Flow cytometric analysis of TLR2 and TLR4 surface expression human SMC

To determine cell-surface TLR expression, 4 × 10^4 ^cells fixed in 3.2% formaldehyde were preincubated in 50 μL of staining buffer (0.5% BSA and 0.1% sodium azide in PBS) containing 10% human serum and 30 μg of human IgG (Sigma) for 30 minutes on ice to block Ig receptors as described previously, [13]. Briefly phycoerythrin-conjugated anti-human TLR2 (clone TL2.1) or TLR4 (clone HTA125), or the relevant phycoerythrin-conjugated isotype control antibodies (IgG2a,κ or IgG1,κ; 0.75 μg each; eBioscience, San Diego, Calif) were then added, and the cells were incubated for a further 60 minutes on ice. A FACScan flow cytometer (Becton Dickinson Immunocytometry Systems [BIDS], Oxford, United Kingdom) was used, and typically 10,000 events were acquired in the viable cell region of the forward light scatter/side light scatter plots. Analysis was performed with CELLQuest software (BIDS). The fluorescence signal was calculated as the geometric mean fluorescence intensity of the gated ASMC population.

### Effect of whole bacteria on Eotaxin-1, CXCL-8, and GMCSF release

Human ASMC were plated at seeding density of 1 × 10^4 ^cells/cm^2 ^onto 96 well plates. Confluent cells were cultured in DMEM containing sodium pyruvate [4], L-glutamine (2 mM), 10% foetal calf serum, penicillin (100 U ml^-1^)/streptomycin (1 μg ml^-1^), amphotericin B (1,5 μg ml^-1^). Cells were treated in triplicate with either medium only or a model organism for Gram-negative bacteria, (*E. coli *10^6^–10^8 ^CFU ml^-1^), or Gram-positive bacteria, (*S. aureus*, 10^6^–10^8 ^CFU ml^-1^l), for 24 or 48 hours (h) at 37°C in an atmosphere of CO_2_. The supernatants were collected and stored at -80°C to measure the levels of eotaxin-1, CXCL-8, and GMCSF release by using DuoSet ELISA kit (R&D Systems) according to the manufacturers' instructions. The samples were diluted until the cytokine level was within the linear range of the standard curve whenever it was required.

### Effect of selective TLR2 and TLR4 ligands on chemokine release

Confluent cells were treated in triplicate with either medium only or with relevant PAMPs for TLR4 (LPS, 0.01–1 μg ml^-1^), TLR2/TLR1 (Pam_3_CSK4), TLR2/TLR6 (FSL-1, 10–1000 ng ml^-1^), NOD1 (FK565, 10 nM and 100 nM) or NOD2 (MDPLys18, 10 nM and 100 nM) for 24 h or 48 h at 37°C in a CO_2 _incubator.

### Effect of whole bacteria or TLR2/TLR4 ligand on IL-1β induced chemokine release

Confluent cells were treated with Gram-negative (*E. coli*, 10^7^–10^8 ^CFU ml^-1^), Gram-positive (*S. aureus*, 10^7^–10^8 ^CFU ml^-1^), LPS (0.01–1 μg ml^-1^l) or FK565, 10 nM and 100 nM)I in the presence and absence of IL-1β (1 ng ml^-1^) for 24 h before chemokines were measured in the supernatant as described above.

### Effect of selective TLR3 ligand Poly I:C on chemokine release

Cells were treated in triplicate with either medium only or with Poly I:C (0.01 -10 μg/ml) alone or Poly I:C (1 or μg/ml) in the presence of *E. coli *(10^6 ^CFU ml^-1^), *S. aureus *(10^6 ^CFU ml^-1^) or LPS at 0.01 μg ml^-1 ^for 24 h before chemokine release was measured as described earlier.

### Cell number and cell viability

The cell viability was assessed by the mitochondrial-dependent reduction of MTT to formazan on the remaining cells following all treatments mentioned above. Briefly, following the removal of supernatant, the remaining cells were incubated with 1 mg ml^-1 ^MTT in serum free medium at 37°C for 15 min. The MTT was removed and DMSO was added. The OD was read at 550 nm.

### Data and Statistical Analysis

Data are presented as mean ± SEM. Data were compared using one-way analysis of variance (ANOVA) followed by Newman-Keuls post-hoc t-test to determine statistical differences after multiple comparisons using Prism software. A probability value of less than 0.05 was considered significant.

## Results

### Effect of Gram-negative *E. coli *and associated PAMPs on release of CXCL-8

**Figure 1:**
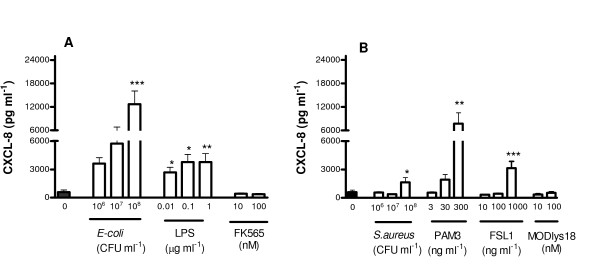
**Effect of bacteria and associated PAMPs on CXCL-8 release from human ASMCs over 24 h.** Panel A, human ASMCs treated with Gram-negative *bacteria*, LPS or FK565. Panel B, cells treated with Gram-positive bacteria, Pam_3_CSK4, FSL-1, MDPLys18. Data represent mean ± SEM for triplicate of three different donors. * p < 0.05; **p < 0.01; ***p < 0.001 compared with un-stimulated control (medium only).

*E. coli *activates PRRs including TLR4 and NOD1. LPS is a PAMP for TLR4 and FK565 is a PAMP for NOD1. Under control culture conditions, human ASMCs released relatively low, but detectable levels of CXCL-8. When *E. coli *(10^6 ^to 10^8 ^CFU ml^-1^) was added to human ASMCs, the release of CXCL-8 was increased at 24 (Figure 1) or 48 hours (table 1). In line with this, cells stimulated with LPS (0.01 to 1 μg ml^-1^) released increased levels of CXCL-8 (Figure 1; table 1). By contrast, FK565 (10 to 100 nM), did not affect CXCL-8 release when administered alone (Figure 1). In separate experiments, it was noted that FK565 had no interaction with LPS in the release of CXCL-8 from human ASMCs (control, 48.39 ± 17.9; plus LPS, 161.7 ± 21.9; plus FK565, 23.07 ± 8.3 LPS+FK565, 123.9 ± 17.3, n = 3).

**Table 1:** 

**Treatments**	**Concentrations**	**Mean ± s.e.m**
control	0	298 ± 71.17
*E. coli *(CFU ml^-1^)	10^6^	4566 ± 1218
	10^7^	7060 ± 1866
	10^8^	17130 ± 5373

LPS (μg ml^-1^)	0.01	2500 ± 739.5
	0.1	4392 ± 1131
	1	4217 ± 1050

FK565 (10 nM)	10	177.3 ± 88.16
	100	150.8 ± 67.31

Effect of Gram-negative bacteria and associated PAMPs on CXCL-8 release from human ASMCs over 48 h. ASMCs were treated with either bacteria Gram-negative *E. coli*, (10^6^–10^8 ^CFU ml^-1^), LPS, (0.01–1 μg ml^-1^) or FK565, (10 nM and 100 nM). The data represent the mean ± SEM from triplicate of three different donors.

### Effect of Gram-positive *S. aureus *and associated PAMPs on release of CXCL-8 from human ASMCs

*S. aureus *activates PRRs including TLR2/TLR1, TLR2/TLR6 and NOD2. Pam_3_CSK4, FSL-1 and MDPLys18 are PAMPs for TLR2/TLR1, TLR2/TLR6 and NOD2 respectively. *S. aureus *(10^6 ^to 10^8 ^CFU ml^-1^) stimulated the release of CXCL-8 from human ASMCs at 24 (Figure 1) and 48 h (table 1). Similarly Pam_3_CSK4 (3 to 300 ng ml^-1^) or FSL-1 (10 to 1000 ng ml^-1^) increased the release of CXCL-8 at 24 (Figure 1) and 48 h (table 2). By contrast, MDPLys18 (10 to 100 ng ml^-1^) had no significant effect on the release of CXCL-8 (Figure 1).

**Table 2:** 

**Treatments**	**Concentrations**	**Mean ± s.e.m**
control	0	248.3 ± 76.44
*S. aureus *(CFU ml^-1^)	10^6^	328.8 ± 132.1
	10^7^	170.0 ± 56.09
	10^8^	2564 ± 1062

PAM 3 (ng ml^-1^)	3	405.3 ± 103.0
	30	2108 ± 531.5
	300	7994 ± 2178.0

FSL1 (ng ml^-1^)	10	370.6 ± 144.4
	100	509.0 ± 214.4
	1000	3792 ± 1379

MDPLys18 (10 nM)	10	218.1 ± 95.11
	100	444.1 ± 143.1

Effect of Gram-positive bacteria and associated PAMPs on CXCL-8 release from human ASMCs over 48 h. ASMCs were treated either Gram-positive bacteria *S. aureus*, (10^6^–10^8 ^CFU ml^-1^), Pam_3_CSK4 (3 – 300 ng ml^-1^), FSL-1, (10 – 1000 ng ml^-1^) or MDPLys18 (10 nM and 100 nM). The data represent the mean ± SEM of triplicate of three different donors.

### Effect of Gram-negative *E. coli *and associated PAMPs on release of eotaxin-1 from human ASMCs

**Figure 2:**
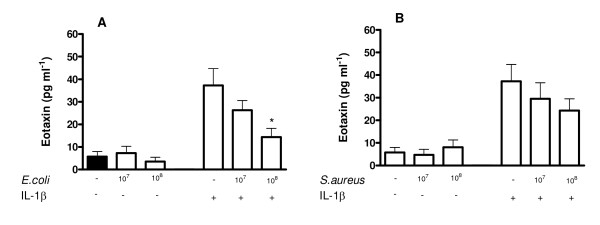
**Effect of Gram-negative or Gram-positive bacteria on eotaxin-1 release in human ASMC over 24 h.** ASMCs were treated with *E. coli *(panel A), or *S. aureus *(panel B) in the present or absent of IL-1β (1 ng ml^-1^). Data are mean ± SEM.; from triplicate of three different donors. * p < 0.05 compared with control (cell stimulated with IL-1β only).

Under control culture conditions, ASMCs released relatively low or undetectable levels of eotaxin-1. When *E. coli *(10^6 ^to 10^8 ^CFU ml^-1^) was added to human ASMCs, in experiments from cells of donors where 'basal' levels were detectable, a reduction in 'basal' release was noted (data not shown). In subsequent experiments, cells were stimulated with IL-1β in order to induce eotaxin-1 release and the effects of co- treatment with bacteria or PAMPs were studied. *E. coli *induced a concentration dependent reduction in the release of eotaxin-1 stimulated with IL-1β. This was significant at 10^8 ^CFU ml^-1 ^(Figure 2). Neither LPS, nor FK565 given alone or in combination affected the release of eotaxin-1 induced by IL-1β (data not shown).

### Effect of Gram-positive *S. aureus *and associated PAMPs on release of eotaxin-1 from human ASMCs

Similar to observations with *E. coli*, *S. aureus *(10^7 ^and 10^8 ^CFU ml^-1^) did not induce the release of eotaxin-1 from human ASMCs (Figure 2). Furthermore, when cells were co-stimulated with IL-1β, *S. aureus *tended to reduce eotaxin-1 release, although this did not reach statistical significance (Figure 2). In line with observations from whole bacteria, neither Pam_3_CSK4 nor FSL-1 nor MDPLys18 affected eotaxin-1 release by human ASMCs (data not shown).

### Effect of Gram-negative *E. coli *or Gram-positive *S. aureus *bacteria on GMCSF release by human ASMCs

**Figure 3:**
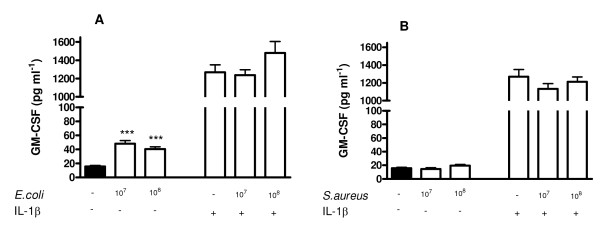
**Effect of bacteria on GMCSF release from human ASMCs over 24 h.** ASMCs treated with Gram-negative bacteria, *E. coli*, (panel A), or Gram-positive bacteria *S. aureus *(panel B) in the presence or absence of IL-1β (1 ng ml^-1^). The data represent the mean ± SEM of triplicate of three different donors. ***p < 0.001 compared with un-stimulated control (medium only).

Under control culture conditions, human ASMCs released low or undetectable levels of GMCSF. Stimulation of cells with *E. coli *(10^7 ^and 10^8 ^CFU ml^-1^) induced increased release of GMCSF (Figure 3). However, *S. aureus *(10^7 ^or 10^8 ^CFU ml^-1^) had no effect on the release of GMCSF (Figure 3). In the presence of IL-1β, GMCSF release was increased. Neither *E. coli *nor *S. aureus *had any additional effect on GMCSF release in cells stimulated with IL-1β (Figure 3).

### Effect of Poly I:C on chemokine release by human ASMCs

**Figure 4:**
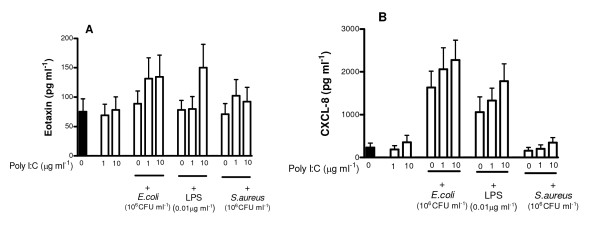
**Effect of selective Poly I:C on chemokine release from human ASMC.** ASMCs were treated in triplicate for 24 h with either medium only or with TLR3 (Poly I:C) alone or in the present of Gram-negative bacteria, *E. coli*, or Gram-positive bacteria, *S. aureus *or LPS (Panels A&B). The data represent the mean ± SEM of three different donors.

Viral PAMPs activate intracellular PRRs. Poly I:C is a synthetic form of viral dsRNA and a selective ligand for TLR3. Poly I:C (0.01 -10 μg/ml) had no effect on the release of CXCL-8 or eotaxin-1 from human ASMCs stimulated for 24 h (data not shown). When cells were co-treated with Poly I:C and LPS, *E. coli *or *S. aureus *there was a tendency, which did not reach statistical significance, for eotaxin-1 and CXCL-8 to be increased (Figure 4).

### Expression of TLR2 and TLR4 by human ASMCs

As we have shown previously [13], human ASMCs express detectable levels of TLR2 or TLR4 (Table 3). Incubation of cells with whole Gram-positive or Gram-negative bacteria did not significantly alter levels of TLR expression (Table 3).

**Table 3:** 

**Treatments**	**TLR2 (MFIr)**	**TLR4 (MFIr)**
control	1.34 ± 0.1	1.81 ± 0.1
*E. coli*	1.4 ± 0.03	1.66 ± 0.1
*S. aureus*	1.3 ± 0.05	1.59 ± 0.04

Flow-cytometric analysis of cell surface TLR2 and TLR4 protein in human ASMC. Cells were treated with *E. Coli or S. Aureus *(10^8 ^CFU/ml) for 48 hrs. Data is expressed as ratio of mean fluorescence intensity (MFIr) of cells stained with specific TLR2 or TLR4 antibodies and cells stained with the relevant isotype control antibodies. The data is the mean ± SEM of n = 3–4 ASMC donors.

## Discussion

In the current study we demonstrate that human ASMCs respond directly to Gram-negative or Gram-positive bacteria. We also show that there is a differential relationship between the release of CXCL-8 and eotaxin-1 by these cells when stimulated with bacteria.

Gram-negative *E. coli *or Gram-positive *S. aureus *activated human ASMCs to release CXCL-8. CXCL-8 is a potent chemoatractant for neutrophils. Recruitment of neutrophils is an early event at the site of bacterial infection and these observations identify an innate immune function of ASMCs which may be relevant in conditions such as asthma or COPD.

In most cells, the effects of Gram-negative bacteria can be mimicked by LPS, which activates TLR4 [15]. Gram-negative bacteria also contain peptidoglycan which can activate NOD receptors. The effect of *E. coli *on CXCL-8 by human airway smooth muscle cells was mimicked by LPS, but not by FK565, which activates NOD1 receptors. In some cell types LPS synergizes with FK565 to activate cells [16,17]. However, in the current study using ASMCs, we found no evidence for interaction between these two pathways. These observations suggest that the ability of Gram-negative *E. coli *to stimulate airway smooth muscle cells to release CXCL-8 is mediated by TLR4 and not NOD1 receptor activation.

Gram-positive bacteria contain PAMPs for TLR2, which can heterodimerize with either TLR1 or TLR6 [18]. In our hands, activation of either the TLR2/TLR1, with Pam_3_CSK4 or TLR2/TLR6, with FSL-1 resulted in a stimulation of ASMCs and release of CXCL-8. Peptidoglycan from Gram-positive bacteria activates mainly NOD2 [16,19]. MDPLys18 which is a selective ligand for NOD2, had no effect on CXCL-8 release by airway smooth muscle cell; these observations suggest that the effects of *S. aureus *on airway smooth muscle are mediated by TLR2 and not by NOD receptor activation.

Interestingly, by contrast to results with CXCL-8, we found that *E. coli*, but not *S. aureus*, increased the release of GMCSF by human airway smooth muscle cells. In other cells, the effects of *E. coli *are mediated principally by TLR4 and those of *S. aureus *by TLR2 [20]. TLR4 recruits both TRIF/TRAM and MyD88/MAL adapter protein pathways. TLR2, on the other hand, recruits MyD88/MAL only. It may well be, therefore, that CXCL-8 is a MyD88/MAL-dependent gene whereas GMCSF, like M-CSF [21], or NOSII [22,23], is independent of the MyD88 pathway and is mediated by TRIF/TRAM.

By contrast to results obtained with CXCL-8, we found that bacteria did not stimulate the release of eotaxin-1. In fact, in cells co-treated with IL-1β, Gram-negative *E. coli *inhibited eotaxin-1 release. A similar trend was seen for cells stimulated with IL-1β and *S. aureus*. If the observations we make here with cells *in vitro *translate to the *in vivo *situation, we may expect bacteria to reduce eotaxin-1 in the airways, limit the numbers of eosinophils recruited and reduce the associated respiratory symptoms in allergic airway disease. Interestingly, recent studies have shown that bacterial DNA immunotherapy can successfully inhibit eosinophil recruitment into the airways in models of allergic lung disease [24]. In the current study, we found that the inhibitory effects of *E. coli*, on eotaxin-1 release were not mimicked by LPS or by FK565 in our experimental conditions. Whilst not tested directly, our observations are consistent with the effects of *E. coli *on eotaxin-1 release being mediated by DNA and not by TLR4 or NOD receptors. Our data shows that treatment of cells with Poly:IC did not induce CXCL-8 or eotaxin release. We have shown previously that Poly I:C at a concentration of 25 μg/ml induced the release of eotaxin and IL-8 from airway smooth muscle cells [13], but in the current study, we did not find an effect of Poly I:C at 10 μg/ml although eotaxin release has been reported at this concentration [25]. This may be due to the fact that we treated the cells under different conditions, that is, in the presence of serum, while the other studies used serum-free conditions.

It is important to note that the observations we report here may well have important implications for other respiratory conditions in which bacterial infections are prevalent such as cystic fibrosis. In cystic fibrosis, colonisation of the lungs with bacteria is known to contribute to exacerbations. Moreover, remodelling of airway smooth muscle in this condition [26-28] may lead to an alteration in the balance of chemokine release in response to bacteria.

## Conclusion

We show that whole Gram-positive or Gram-negative bacteria activate human airway smooth muscle cells to release CXCL-8. Using selective PAMPs, we establish that the effects of whole bacteria on CXCL-8 are likely to be mediated by TLRs and not by NOD receptors. By contrast to CXCL-8, we found that bacteria inhibited the release of eotaxin-1 from human ASMCs. In the case of eotaxin-1 release, the effects of Gram-negative bacteria did not appear to be mediated by TLR4 or NOD pathways. These observations establish a clear but complex innate immune function of ASMCs with regard to bacterial infections.

## Abbreviations

ASMC: airway smooth muscle cells, *E. coli*: *Escherichia coli*, ELISA: Enzyme-linked immunosorbent assays, IL-1β : Interlukin-1β, LPS: lipopolysaccharide, MTT: (3- [4,5-dimethylthiazol-2-yl]-2,5-diphenyltetrazolium bromide), PAM3: Pam_3 _Cys-Ser-(Lys)4, *S. aureus*, *Staphylococcus aureus*, TLR: Toll-like receptors

## Competing interests

The author(s) declare that they have no competing interests.

## Authors' contributions

RI is the first author to this study and was responsible for the overall collation of the work and had the drive to initiate and complete the study. RI and RS participated in the design and coordination of the study, analyzed the results and helped in drafting the paper. They also contributed to the drafting of the revised manuscript. MS contribution came after the initial review from the journal and included the design, execution and analysis of the experiments showing TLR expression by FACS analysis. MS also contributed to the drafting of the revised manuscript and control data showing that anti-TLR antibodies are unreliable tools for the specific block of responses, this information was included in the response to reviewers. SS provided expert advice and guidance for the overall project, particularly the relevance of this work to the field and was also specifically responsible for all information and concepts relating to the usage of whole bacteria. JAM worked closely with RI at the initial stages and throughout the course of this study. JAM, together with RI, was responsible for the initial draft of the manuscript and design of the basic study. KFC conceived of the study in general terms with JAM and RI. KFC participated in its design and coordination, helped with interpretation of data and the drafting of the manuscript. All authors have read and approved the manuscript.
